# Regional differences in reasons for consultation and general practitioners’ spectrum of services in northern Germany – results of a cross-sectional observational study

**DOI:** 10.1186/s12875-020-1093-6

**Published:** 2020-01-31

**Authors:** Ingmar Schäfer, Heike Hansen, Thomas Ruppel, Dagmar Lühmann, Hans-Otto Wagner, Agata Kazek, Martin Scherer

**Affiliations:** Department of Primary Medical Care, University Medical Center Hamburg-Eppendorf, Martinistr. 52, 20246 Hamburg, Germany

**Keywords:** General practice; Healthcare utilisation, Reasons for consultation, Regional comparison, Urban-rural differences

## Abstract

**Background:**

Among other factors, the patients’ consultation reasons and GPs’ spectrum of services determine the process and outcome of the medical treatment. So far, however, there has been little information on differences in reasons for consultation and GPs’ services between urban and rural areas. Our study’s goal was thus to investigate these factors in relation to the regional location of GPs’ practices.

**Methods:**

We conducted a cross-sectional observational study based on standardised GP interviews in a quota sampling design. All counties and independent cities within a radius of 120 km around Hamburg were divided into three regional categories (urban area, environs, rural area) and stratified proportionally to the population size. Differences in the number of reasons for consultation and services were analysed by multivariate linear regressions in mixed models adjusted for random effects on the levels of the German federal states and administrative districts. Differences in individual consultation reasons and services were identified by logistic regression via stepwise forward and backward selection.

**Results:**

Primary care practices in 34 of the 37 selected administrative districts (91.9%) were represented in the dataset. In total, 211 GPs were personally interviewed. On average, GPs saw 344 patients per month with a slightly higher number of patients in rural areas. They reported 59.1 ± 15.4 different reasons for consultation and 30.3 ± 3.9 different services. There was no statistically significant regional variation in the number of different consultation reasons, but there was a broader service spectrum by rural GPs (ß = − 1.42; 95% confidence interval − 2.75/− 0.08; *p* = 0.038) which was statistically explained by a higher level of medical training. Additionally, there were differences in the frequency of individual consultation reasons and services between rural and urban areas.

**Conclusion:**

GPs in rural areas performed more frequently services usually provided by medical specialists in urban areas. This might be caused by a low availability of specialists in rural areas. The association between medical training and service spectrum might imply that GPs compensate the specific needs of their patients by completing advanced medical training before or after setting up a medical practice.

**Trial registration:**

The study was registered in ClinicalTrials.gov (NCT02558322).

## Background

In well-developed health care systems, there is rational health care planning based on available data. However, not all relevant data are normally used for this process. In Germany, for example, GPs are mostly self-employed in independent small businesses. They can work individually in private practices (individual practice) or together with other physicians – either in private practices where each physician submits his own claims (group practice) or in private practices where the claims of all physicians are combined and submitted as one bill (joint practice). It is also possible that GPs work as employees or self-employed in medical care centres (“*Medizinische Versorgungszentren*”). In the statutory health insurance system, which includes approximately 90% of the German population, about 95% of the services are determined by the Federal Joint Committee. The remainder of the services can be defined individually by the statutory health insurance companies based on strict legal requirements [1].

The regional associations of statutory health insurance physicians and the regional federations of the statutory health insurances are obliged to perform mutual needs-related planning of the number of accredited physicians within the statutory health insurance system. In order to prevent an over- and undersupply of physicians, ratios of the number of physicians in different specialties and the population size in specific planning areas are defined. These ratios can be adapted to special features of the region or the regional infrastructure, especially if other planning areas contribute to health care in the respective area. Other criteria for deviating from these ratios are regional data on morbidity, sociodemographic parameters or socioeconomic status of the population [2].

On the one hand, according to these criteria, there are substantial regional disparities. For example, diabetes and obesity are more prevalent in rural areas [3] while depressive disorders play a slightly larger role in core cities [4]. Age groups differ largely between East and West Germany as well as between urban and rural areas [5]. Additionally, from a socioeconomic perspective, there are inconsistent regional differences between urban and rural areas. For example, while highly qualified jobs are concentrated in the centres of urban areas [6], there is also simultaneously a significantly higher spending capacity adjusted income poverty in urban areas [7].

On the other hand, not all factors relevant for the utilisation of health care are covered by these criteria. For example, barriers and enablers for access to health care differ between the regions. The preliminary qualitative study of the analysis presented here reported that GPs and patients in rural areas frequently discussed long distances to the doctor’s office, long waiting times and the GPs’ large workload. In contrast, high patient fluctuation and fierce competition among GPs were frequent topics of discussions in urban areas [8].

There were also regional differences in patient behaviour and GPs’ understanding and perception of their role. Patients in rural areas seemed to consult their GP relatively late in urgent consultation matters, while a certain fraction of the patients in cities already consulted their GP for banalities [8]. Furthermore, GPs in rural areas were more likely to identify themselves as family doctors and persons of trust for their patients while their role as medical service provider and referrer to specialists was more emphasised in urban areas [9].

These aspects are very likely linked to regional variations of patients’ reasons for consultation and GPs’ spectrum of services, which also determine process and outcome of the medical treatment. A study by Steinhäuser et al. showed clear differences between rural and urban GPs regarding 25 of the 42 most frequently carried out procedures (such as “ambulatory blood pressure monitoring” or “adolescent examinations”) [10]. So far, however, there has been relatively little information on the difference in reasons for consultation and services between urban and rural areas. Thus, the goal of the study presented here was to investigate reasons for consultation and services of GPs in relation to the regional location of their practices in Northern Germany.

## Methods

The project “Outpatient Healthcare Research North (*Ambulante Versorgungsforschung Nord - AVFN*)” was designed as a cross-sectional observational study. Methods, research hypotheses and statistical models had been described in the study register ClinicalTrials.gov (NCT02558322) before starting the survey and in the published study protocol [11]. Although our study has been based on GP and patient interviews, the analysis presented here includes GP data only.

Three categories had been defined for the regional comparison according to the so-called “structural settlement of district types” of the German Federal Institute for Research on Building, Urban Affairs and Spatial Development [12]. The category “urban areas” included urban municipalities, the category “environs” urbanised districts and rural districts with signs of agglomeration, and the category “rural areas” sparsely populated rural districts.

A quota sampling design was chosen to represent as many regionally different healthcare situations in the study area as possible. The purpose of this design was to raise the probability of also including underserved regions into the study where usually many GPs were unwilling to participate in a study due to their heavy workload. The goal was to represent as many individual administrative districts of the survey area as possible. We stratified the sample a) by regional category based on a pre-defined sample size of at least 80 GPs in each category and b) within the regional categories by counties and independent cities proportionally to the respective population size in each district. In each regional category, a 25% maximum deviation from the recruitment plan was accepted [11].

At first and for determining the survey area, all administrative districts were included in the study where at least 20% of the land area was located within a radius of 100 km (ca. 62 miles) linear distance around the study centre (University Medical Centre Hamburg-Eppendorf). As initially, despite regular follow-up actions, the desired sample size of 240 GPs could not be achieved, the radius around the study centre was expanded to 120 km (ca. 75 miles) according to the approach described in the study protocol [11]. The thus chosen administration districts for the study were derived from the German Federal States of Bremen, Hamburg, Mecklenburg-Western Pomerania, Lower Saxony, Saxony-Anhalt and Schleswig-Holstein.

GPs’ recruitment was based on a database of the Department of General Practice at the University Medical Centre Hamburg-Eppendorf as well as on the databases of the respective regional associations of statutory health insurance physicians in the selected areas. GPs were eligible for the study if they had an accreditation as statutory health insurance physician in the respective administrative district and used an EDP system facilitating to draw up a list of all patients treated over the preceding quarter (3-month accounting period).

Eligible GPs were contacted in writing by mail and asked to participate in the study. Participating GPs were visited by a staff member of the project and personally interviewed. In rare cases, it had been impossible to completely finish the GP’s interview during the office visit for lack of time. Regarding these cases, the interview was later completed by telephone. Data were supplied by memory recall. However, GPs were allowed to check their medical records if they considered it necessary.

The interviews were based on standardised questionnaires and contained information regarding the GPs’ age (1 item), gender (1 item), workload (3 items), and postgraduate and advanced medical training (2 items) as well as data on the practice (4 items). Additionally, we documented 99 different reasons for consultation from 17 areas / organ systems (“How often (per day/week/month/year, rarely, never) do you see patients with the following reasons for consultations (including home visits)?”) and healthcare services involving 38 different procedures (“How often (per day/week/month/year, rarely, never) do you provide the following services (including home visits)?”). This assessment was based on a standardised instrument developed on the basis of the International Classification of Primary Care (ICPC-2) [11, 13, 14]. The resulting data were described as the proportion of the respective consultation reasons or the respective services of all consultation reasons or services documented at the respective practice. The GP questionnaire also contained some items not reported here, ie, the GPs’ place of residence (1 item) and professional experience (4 items) as well as the number of weekly contacts with 27 patient types. The GPs’ survey was conducted from 12 June 2015 to 27 April 2017.

Regional differences were initially described using the chi-square test and t-test. The number of different consultation reasons and the GPs’ service spectrum were subsequently analysed by means of multivariate linear regressions in mixed models adjusted for random effects on the levels of the German federal states and administrative districts within the federal states. In these analyses, the control variables were stepwise included in three statistical models. A potential improvement of the model fit was determined by the likelihood ratio test.

To be able to interpret these results better, significant differences in reasons for consultation and services between urban areas, environs, and rural areas were identified by logistic regression analyses via stepwise forward and backward selection with *p* ≤ 0.05 as inclusion and *p* ≤ 0.10 as exclusion criterion. Variables showing characteristic values of a lower frequency than 1% had been excluded in advance. Thus, 14 ICPC 2 areas / organ systems, 29 different reasons for consultation and 22 different services remained in the statistical models for selecting variables.

An alpha level of 5% (*p* ≤ 0.05) was defined as statistically significant for all analyses of the inferential statistics. Stata 15.1 was used for data preparation and data analysis.

## Results

**Fig. 1:**
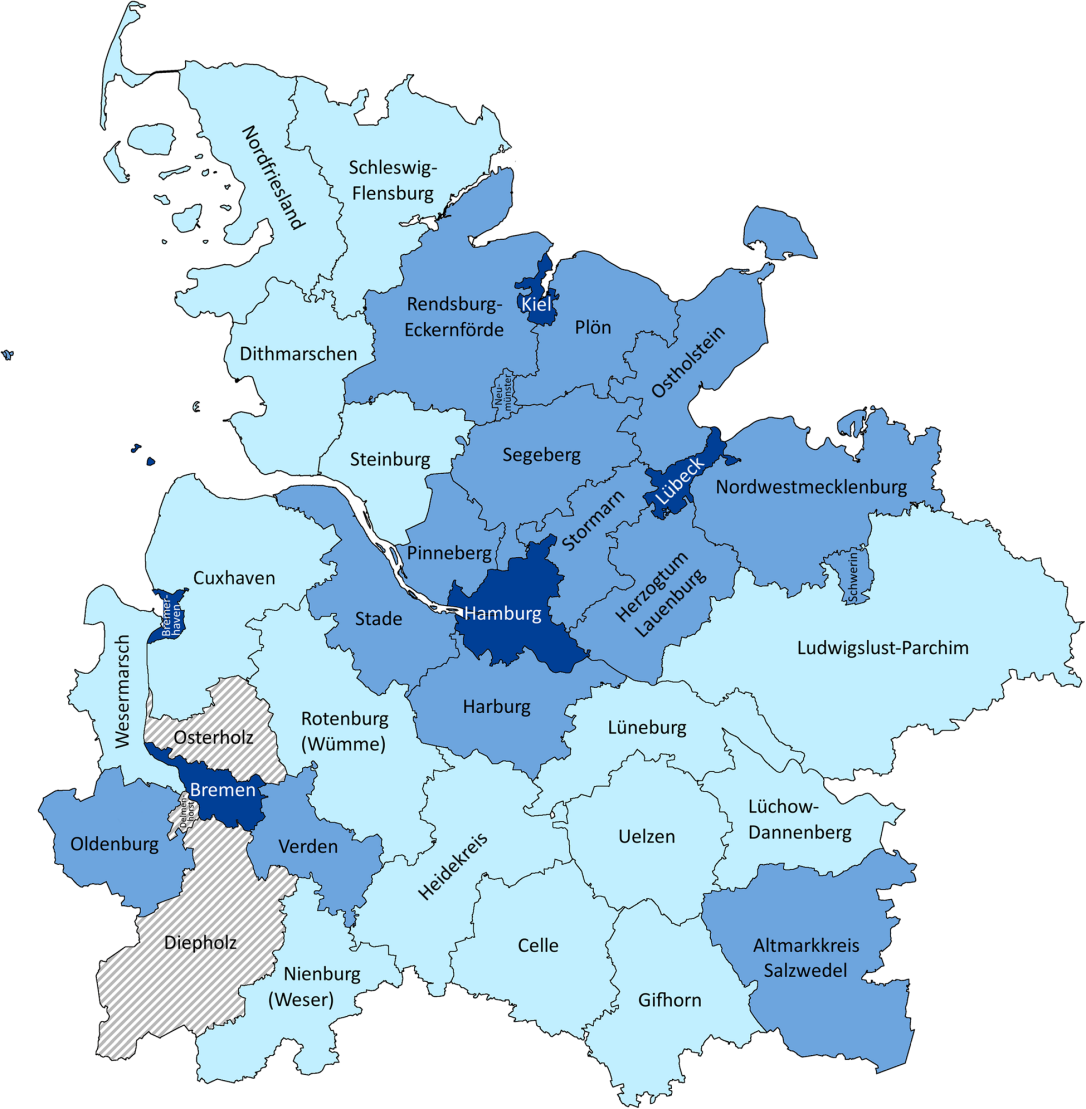
Included administration districts with regional categories.© GeoBasis-DE / German Federal Agency for Cartography and Geodesy (BKG) 2017 (adjusted). Legend: light blue areas – administration districts of the category “rural areas”; medium blue areas – administration districts of the category “environs”; dark blue areas – administration districts of the category “urban areas”; grey and white shaded areas – not represented administration districts

In our quota sampling design, we were able to adhere to the stratification rules defined in the study protocol. Figure 1 shows a map of the respective regions. Primary care practices in 34 of the 37 selected administrative districts (91.9%) were represented in the dataset. No study participants could be recruited from three districts of the environs, ie, Delmenhorst, Diepholz and Osterholz. Initially, we recruited 280 GPs from the selected regions. However, interviews could not be carried out with 69 study participants due to time-related or organisational reasons (eg, sick primary care partners, problems with the patient management software). In the end, we were able to personally interview 211 GPs. Tables S1 to S3 of the Additional file 1 describe the recruitment process of the stratification groups. In total, 12% of GPs in urban areas, 13% of GPs in the environs, and 25% of GPs in rural areas had to be recruited from other stratification groups of the respective regional category than originally planned.

The sociodemographic data, the number of treated patients, and the professional qualification of the interviewed GPs are shown in Table 1. The GPs’ average age was 54.5 years and 65.4% of GPs were male. On average, GPs saw 344 patients per month with a slightly higher number of patients in rural than in urban areas. In our study, GPs mostly worked in individual or joint practices and less frequently in group practices or medical care centres. Individual practices were noticeable more frequently found in rural than in urban areas and in our sample, group practices and medical care centres had been solely located in urban areas and the environs.

**Table 1:** Sociodemography, number of treated patients and professional qualification

	Total	Urban areas	Environs	Rural areas	p (C/R)	p (O/R)
Age (in years)	54.5 ± 8.6 (*n* = 207)	53.5 ± 7.8 (*n* = 66)	54.7 ± 8.6 (*n* = 72)	55.4 ± 9.2 (*n* = 69)	0.190	0.630
Gender						
female	34.6%	45.5%	27.0%	32.4%	0.117	0.479
male	65.4% (*n* = 211)	54.6% (n = 66)	73.0% (*n* = 74)	67.6% (*n* = 71)		
***Number of patients per month***	344 ± 115 (n = 207)	***314 ± 101 (n = 65)***	345 ± 96 (n = 74)	***372 ± 140 (n = 68)***	***0.007***	0.172
***Type of medical practice***						
***individual practice***	51.7%	***43.9%***	51.4%	***59.2%***	***0.004***	0.074
***group practice***	6.2%	***12.1%***	6.8%	***–***		
***joint practice***	40.8%	***39.4%***	41.9%	***40.9%***		
***medical care centre***	1.4% (n = 211)	***4.6%*** (n = 66)	– (n = 74)	***–*** (n = 71)		
***Medical specialist training***						
none	6.6%	9.1%	8.1%	2.8%	0.118	0.163
***general practice***	77.3%	77.3%	***70.3%***	***84.5%***	0.281	***0.041***
***internal medicine***	23.2%	25.8%	***31.1%***	***12.7%***	0.051	***0.008***
others	3.8% (n = 211)	1.5% (n = 66)	8.1% (n = 74)	1.4% (n = 71)	0.959	0.060
Number of areas of advanced medical training	1.5 ± 1.6 (n = 211)	1.2 ± 1.4 (n = 66)	1.7 ± 1.7 (*n* = 74)	1.5 ± 1.8 (*n* = 71)	0.268	0.498
Advanced medical training						
none	31.8%	40.9%	25.7%	29.6%	0.165	0.599
urgent and emergency care	24.2%	19.7%	27.0%	25.4%	0.429	0.819
palliative medicine	17.1%	9.1%	20.3%	21.1%	0.051	0.899
acupuncture	12.3%	13.6%	9.5%	14.1%	0.940	0.387
manual medicine	12.3%	7.6%	17.6%	11.3%	0.461	0.281
natural medicine	12.3%	13.6%	13.5%	9.9%	0.492	0.494
sports medicine	7.1%	3.0%	12.2%	5.6%	0.457	0.169
nutritional medicine	6.6%	3.0%	12.2%	4.2%	0.709	0.083
addiction basic care	6.6%	7.6%	5.4%	7.0%	0.905	0.683
diabetology	6.2%	4.6%	8.1%	5.6%	0.773	0.557
geriatrics	6.2%	4.6%	9.5%	4.2%	0.927	0.214
psychotherapy	6.2%	7.6%	5.4%	5.6%	0.647	0.952
drivers medical assessment	6.2% (n = 211)	7.6% (n = 66)	5.4% (n = 74)	5.6% (n = 71)	0.647	0.952

*C/R* comparison “urban areas” vs. “rural areas”, *O/R* comparison “environs” vs. “rural areas”

Statistically significant results (*p* ≤ 0.05) are shown in bold and italic

77.3% of GPs had completed general medicine postgraduate specialist training and 23.2% had completed internal medicine postgraduate specialist training. The postgraduate training “general medicine specialist” was reported more often in rural areas than in the environs and the postgraduate training “internal medicine specialist” was reported less often in rural areas than in the environs. 6.6% of GPs reported that they had not completed a postgraduate specialist training. On average, GPs additionally reported advanced medical training in 1.5 areas. The most frequent advanced medical training was in emergency care and palliative medicine. 31.8% of GPs had not completed an advanced medical training.

Table 2 shows the average relative rate of individual consultation reasons of all reported reasons for consultation. Most frequently, GPs described reasons for consultation from the ICPC 2 areas / organ systems “cardiovascular system”, “musculoskeletal system” and “endocrine/metabolic and nutritional”. In total and on average, GPs reported 59.1 ± 15.4 different reasons for consultation that had been addressed by their patients during consultation at least once per month.

**Table 2:** Relative frequency of the most prevalent reasons for consultation from the GP’s perspective

	Total (*n* = 201)	Urban areas (*n* = 65)	Environs (*n* = 71)	Rural areas (*n* = 65)	p (C/R)	p (O/R)
Cardiovascular system, such as:	14.6%	14.8%	14.8%	14.3%	0.737	0.749
high blood pressure	6.7%	6.8%	6.8%	6.4%	0.661	0.639
coronary heart disease	2.6%	2.6%	2.8%	2.5%	0.682	0.355
cardiac arrhythmia	2.0%	2.1%	1.9%	1.9%	0.653	0.959
congestive heart failure	1.9%	1.7%	1.8%	2.1%	0.169	0.356
***Musculoskeletal system, such as:***	12.4%	***10.5%***	12.8%	***13.8%***	***0.001***	0.330
***spinal disorders***	6.7%	***5.4%***	7.5%	***7.0%***	***0.006***	0.487
***arthrosis of the large joints***	1.7%	1.7%	***1.5%***	***2.0%***	0.289	***0.041***
***shoulder pain***	1.5%	***1.2%***	***1.3%***	***2.0%***	***0.001***	***0.003***
Endocrine/metabolic/nutritional, such as:	12.2%	12.2%	12.5%	11.9%	0.816	0.662
diabetes mellitus (all types)	3.7%	3.5%	3.8%	3.9%	0.304	0.838
lipid metabolism disorder	2.8%	2.6%	3.0%	2.7%	0.891	0.544
weight/dietary problems	2.6%	2.5%	2.8%	2.3%	0.791	0.306
thyroid disorder	1.5%	1.7%	1.4%	1.5%	0.237	0.832
Respiratory system, such as:	11.2%	11.6%	11.8%	10.2%	0.151	0.072
***acute infections of the respiratory tract***	7.9%	7.9%	***8.7%***	***6.9%***	0.269	***0.029***
chronic obstructive pulmonary disease	1.4%	1.6%	1.1%	1.4%	0.449	0.107
asthma	1.0%	1.2%	0.9%	1.1%	0.499	0.395
Psychological disorders, such as:	10.8%	11.7%	10.3%	10.5%	0.206	0.834
somatoform disorders	3.7%	4.0%	3.5%	3.6%	0.471	0.858
mild mental disorders	2.8%	2.7%	2.9%	2.7%	0.887	0.602
dementia	1.6%	1.6%	1.4%	1.9%	0.622	0.290
General/unspecific disorders, such as:	10.2%	10.7%	9.8%	10.2%	0.578	0.697
general fatigue and weakness	3.8%	4.3%	3.5%	3.5%	0.128	0.982
vertigo	2.0%	2.2%	2.0%	1.9%	0.310	0.974
***lymph/leg oedema, chronic wounds***	1.4%	***1.2%***	1.5%	***1.7%***	***0.009***	0.316
adverse drug reaction	1.2%	1.3%	1.1%	1.1%	0.422	0.934
Digestive system, such as:	9.2%	9.1%	8.9%	9.7%	0.514	0.367
total gastrointestinal tract	3.1%	3.1%	3.1%	3.1%	0.803	0.827
upper gastrointestinal tract	2.9%	2.8%	3.0%	3.0%	0.571	0.879
lower gastrointestinal tract	2.2%	2.2%	1.9%	2.4%	0.683	0.129
***Social problems, such as:***	5.0%	***6.4%***	4.6%	***4.1%***	***0.001***	0.407
family, partnership problems	1.3%	1.5%	1.2%	1.1%	0.059	0.555
***workplace issues, unemployment***	1.2%	***1.5%***	1.1%	***0.9%***	***0.001***	0.198
Urological system, such as:	3.2%	3.2%	3.1%	3.4%	0.637	0.420
urinary tract infection	1.4%	1.4%	1.5%	1.3%	0.902	0.382
chronic kidney disease	1.1%	1.2%	0.8%	1.2%	0.869	0.112
Skin	2.8%	2.4%	3.1%	3.0%	0.074	0.759
Neurological system, such as:	2.7%	2.5%	2.8%	2.9%	0.166	0.824
headache	1.8%	1.6%	1.9%	1.9%	0.142	0.777
***Ear***	1.7%	***1.4%***	1.6%	***2.0%***	***0.005***	0.140
Blood, blood-forming org., immune syst.	1.2%	1.3%	1.2%	1.2%	0.544	0.994
***Eye***	1.1%	***0.9%***	1.0%	***1.2%***	***0.044***	0.281

*C/R* comparison “urban areas” vs. “rural areas”, *O/R* comparison “environs” vs. “rural areas”

Statistically significant results (*p* ≤ 0.05) are shown in bold and italic

A multivariate analysis adjusted for age and gender showed no statistically significant regional variation in the number of different consultation reasons (see Table S4 of the Additional file 1). However, the stepwise variable selection showed that reasons for consultation from the areas “ear” (odds ratio (OR) 1.42; *p* = 0.046), “skin” (OR 1.38; *p* = 0.013) and “musculoskeletal system” (OR 1.13; *p* = 0.003) as well as the consultation reason “lymph/lymphoedema in the leg, chronic wounds” (OR 1.78; *p* = 0.006) were more often reported in rural than in urban areas. On the other hand, consultation reasons from the area “social problems” (1/OR 1.19; p = 0.006) were less frequently reported in rural than in urban areas. Additionally, “shoulder problems” (OR 1.50; *p* = 0.005) were reported more often in rural areas than in the environs and “acute infections of the respiratory tract” (1/OR 1.10; *p* = 0.024) were less frequently reported in rural areas than in the environs.

Table 3 shows the average relative rate of the individual services of all reported services. The most frequently reported services were “blood test”, “referral to a specialist” and “continuous care for the chronically ill”. The average number of different services provided was 30.3 ± 3.9. Compared to urban areas, a somewhat broader spectrum of services was reported in rural areas (31.1 ± 3.7 vs. 29.4 ± 3.9; *p* = 0.010).

**Table 3:** Relative frequency of services from the GP’s perspective

	Total (*n* = 211)	Urban areas (*n* = 66)	Environs (*n* = 74)	Rural areas (*n* = 71)	p (C/R)	p (O/R)
***Blood tests***	14.9%	14.3%	***13.8%***	***16.6%***	0.081	***0.033***
Referral to specialist	14.6%	15.3%	13.7%	14.9%	0.810	0.360
Continuous care for the chronically ill	11.9%	11.4%	13.3%	10.9%	0.690	0.067
***Urine analysis***	6.3%	6.5%	***5.5%***	***6.9%***	0.553	***0.024***
***Lifestyle counselling, social counselling***	5.3%	***6.5%***	***5.5%***	***3.7%***	***0.006***	***0.034***
***House calls (also: calls at nursing homes)***	4.7%	***3.7%***	4.7%	***5.6%***	***0.020***	0.303
***Psychosomatic basic care***	4.0%	***5.3%***	3.6%	***3.1%***	***0.001***	0.300
Pain therapy	3.7%	3.1%	3.6%	4.3%	0.148	0.403
Electrocardiogram (ECG)	3.4%	3.7%	3.2%	3.3%	0.352	0.842
Disease management programme (DMP)	3.1%	2.8%	2.9%	3.5%	0.176	0.232
Vaccination	2.8%	3.1%	2.6%	2.8%	0.560	0.642
***Wound dressing/compression/tamponade***	2.7%	***1.9%***	3.0%	***3.1%***	***0.002***	0.657
Nutrition counselling	2.2%	2.4%	2.1%	1.9%	0.198	0.601
Sonography	2.2%	2.2%	2.2%	2.3%	0.803	0.843
***Local injection/infiltration***	2.0%	***1.2%***	2.6%	***2.2%***	***0.006***	0.395
***Check-up 35***	1.9%	***2.2%***	2.0%	***1.6%***	***0.023***	0.155
Pulmonary function test	1.5%	1.5%	1.2%	1.7%	0.437	0.063
Organisation of patient care/support	1.4%	1.6%	1.4%	1.1%	0.079	0.205
Scatoscopy	1.4%	1.7%	1.3%	1.3%	0.238	0.930
Physical therapy	1.3%	0.9%	1.4%	1.5%	0.177	0.792
Skin cancer screening	1.1%	1.2%	1.1%	0.9%	0.163	0.434
Social medical assessments	1.0%	0.9%	1.1%	0.9%	0.840	0.453
Alternative/natural medicine, acupuncture	0.9%	0.8%	1.4%	0.6%	0.673	0.081
Referral to hospital	0.9%	1.0%	0.8%	0.9%	0.442	0.407
Early detection of cancer (others)	0.8%	0.7%	1.0%	0.7%	0.889	0.175
Ambulatory blood pressure monitoring	0.7%	0.7%	0.7%	0.6%	0.461	0.241
Individual health services for direct payer	0.6%	0.6%	0.8%	0.3%	0.073	0.063
Cardiac stress test ECG	0.5%	0.6%	0.4%	0.4%	0.521	0.969
Palliative therapy	0.5%	0.4%	0.4%	0.6%	0.239	0.147
Paediatric services	0.4%	0.3%	0.5%	0.4%	0.528	0.752
Incision/drainage/flushing/aspiration	0.3%	0.2%	0.4%	0.2%	0.780	0.080
Minor surgery	0.2%	0.1%	0.3%	0.2%	0.162	0.200
Ambulatory ECG monitoring	0.2%	0.2%	0.2%	0.3%	0.677	0.167
Emergency call	0.2%	0.3%	0.1%	0.1%	0.071	0.448
***Patient education (structured education)***	0.2%	0.2%	***0.3%***	***0.05%***	0.082	***0.038***
Living will	0.2%	0.2%	0.1%	0.2%	0.639	0.114
***Closure/fixation/sutures/prosthesis***	0.2%	***0.08%***	0.3%	***0.1%***	***0.032***	0.150
***Instr. manipulation/catheter/intubation***	0.1%	***0.04%***	0.2%	***0.1%***	***0.021***	0.406

*C/R* comparison “urban areas” vs. “rural areas”, *O/R* comparison “environs” vs. “rural areas”

Statistically significant results (*p* ≤ 0.05) are shown in bold and italic

This difference was, at first, confirmed by the multivariate multilevel analysis controlled for age and gender (see Table 4). However, after including postgraduate and advanced medical training, the regional difference disappeared and the model fit improved significantly (*p* = 0.021), ie, the second statistical model fit our data better than the first model. In contrast, the additional inclusion of practice type and number of patients treated per month produced neither significant changes in the estimates of the other independent variables nor a further improvement of the model fit (*p* = 0.502). The stepwise selection of variables showed that the service “local injection / infiltration” (OR 1.26; *p* = 0.046) was more frequently reported in rural than in urban areas and the services “check-up 35” (1/OR 1.47; *p* = 0.013), “psychosomatic primary care” (1/OR 1.20; *p* = 0.003) as well as “lifestyle counselling, social counselling” (1/OR 1.11; p = 0.003) were less frequently reported in rural than in urban areas. Additionally, “urine analysis” (OR = 1.12; *p* = 0.028) was more frequently reported in rural areas than in the environs.

**Table 4:** Association between region and the number of different services by general practitioners: results of a multivariate linear regression adjusted for random effects on the levels of German federal states and administrative districts within the federal states (n = 203)

	Model 1		Model 2		Model 3	
	ß (95% CI)	p	ß (95% CI)	p	ß (95% CI)	p
***Region***						
***urban areas*vs.*rural areas***	***−1.42 (−2.75 to − 0.08)***	***0.038***	−0.93 (− 2.34 to 0.48)	0.196	−0.96 (− 2.47 to 0.55)	0.211
environs vs. rural areas	−0.90 (−2.36 to 0.55)	0.225	− 0.55 (− 1.92 to 0.82)	0.431	− 0.64 (− 2.01 to 0.74)	0.365
***Age of the physician (in years)***	***− 0.07 (− 0.13 to − 0.01)***	***0.016***	− 0.04 (− 0.11 to 0.02)	0.228	−0.04 (− 0.10 to 0.02)	0.238
***Gender of the physician: male*vs.*female***	***1.67 (0.58 to 2.77)***	***0.003***	***1.47 (0.40 to 2.54)***	***0.007***	***1.47 (0.39 to 2.56)***	***0.008***
***Postgraduate medical specialist training***						
***none (general practitioner)***			***−3.18 (−6.19 to − 0.17)***	***0.038***	−2.92 (−5.95 to 0.11)	0.059
general medicine			−0.58 (−2.65 to 1.50)	0.584	−0.58 (−2.66 to 1.50)	0.586
internal medicine			−1.37 (−3.25 to 0.52)	0.156	−1.43 (−3.33 to 0.45)	0.135
***Number of areas of advanced medical training***			0.29 (−0.02 to 0.59)	0.066	***0.31 (0.00 to 0.61)***	***0.050***
Type of practice						
group practice vs. individual practice					0.99 (−1.16 to 3.15)	0.367
joint practice vs. individual practice					0.91 (0.18 to 2.01)	0.103
medical care centre vs. individual practice					−0.53 (4.69 to 3.62)	0.802
Number of treated patients (per 100 patients every month)					0.00 (−0.00 to 0.00)	0.978

Statistically significant results (*p* ≤ 0.05) are shown in bold and italic

## Discussion

### Main findings

In our study, GPs from regions of different degrees of urbanisation reported how often their patients had presented specific reasons for consultation and how often GPs had responded with providing different services. In total, the number of different reasons for consulting GPs was similar in urban and rural areas. However, the frequency of individual reasons for consultation had been different. In contrast, GPs from rural areas performed a larger number of different services than GPs from urban areas. Comparable to the analysis of consultation reasons, the frequency of the individual services also differed between the regional categories.

On the one hand, our study suggests that GPs in rural areas spent less time on communicative, preventive, and consultative medicine than GPs in urban areas. On the other hand, GPs in rural areas more often reported performing services that are usually provided by specialists in urban areas. GPs’ postgraduate and advanced medical training seemed to play an important role for the service spectrum offered by GPs. The multivariate models showed that the broader spectrum of services reported by rural GPs was statistically explained by a higher level of medical training.

### Strengths and limitations of the survey

GPs’ recruitment was not carried out by random selection but by quota sampling. By adhering to the 25% maximum deviation from the recruitment plan defined in the study protocol [11] – we were able to include 91.9% of the selected administrative districts into our study as well as GPs from less-favoured areas difficult to reach by means of transport, such as Kalbe (Milde) or Helgoland. By stratifying the sample by the individual counties and independent cities we were able to represent both, medically undersupplied and overserved as well as socioeconomically deprived and undeprived districts in our data set.

Disadvantages of quota sampling were that, in some districts, many GPs had to be contacted to adhere to the stratification plan while in other districts the recruitment process had to end early without follow-up and not all GPs willing to participate could be included in the study. Thus, the high number of 4956 contacted GPs and the comparatively low participation rate of 4.3% interviewed GPs. Despite the fact that – by definition – the participation rate is not important in quota sampling, it may still affect the representativeness of the GP population. However, it should be noted that the response rate is not the only indicator for a selection bias. Regarding our study, we deem quota sampling the preferable method over random sampling to represent the regionally different healthcare situations because we believe the variation between medically underserved and oversupplied districts to be greater than the variation between GPs within the districts.

Additionally, although random selection is a better known and clearly more frequently used method, studies have shown quota sampling not necessarily to be linked to a larger selection bias than random sampling [15]. To gather some information on a possible selection bias, we performed a comparison of the data of study participants in the included regions with the statistics of the German national association of statutory health insurance physicians. This analysis showed, with one exception, only relatively minor deviations. Thus, GPs participating in our study had only been slightly older (urban areas: + 0.9 years; environs: + 0.4 years; rural areas: + 0.6 years) and slightly more often males than the basic study population of the selected districts (urban areas: + 3.6%; rural areas: + 3.6%). A more distinct selection bias was found regarding the GPs’ gender in the environs where the proportion of men compared to the basic study population had increased by 15.6 percentage points [16].

Besides the uneven gender distribution, there is one more aspect of the regional category “environs” that needs to be discussed. “Environs”, by definition, include urbanised districts and rural districts with signs of agglomeration. However, our data suggest that, depending on the analysed item, features of primary care in the environs are either similar to “urban areas” or similar to “rural areas” (eg, the rate of the consultation reason “social problems” is similar to rural areas while the rate of the service “lifestyle counselling, social counselling” is comparable to urban areas). Therefore, “environs” should be considered a mixed category and findings regarding this regional category need to be interpreted with care.

The statistical methods provided additional strength by considering potential confounders and the cluster structure of the dataset. Aside from the two multivariate analyses on the range of reasons for consultation and services used for analysing the main question of this study, the stepwise variable selection also identified regional differences in the individual reasons for consultation and services. It should be noted that this approach reacts sensitively to differences in the distribution of the variables. The identified model thus might not necessarily be the best set of variables to describe the regional differences in individual services and consultation reasons. As the explained variance had been relatively low (the pseudo-R^2^ averaged 0.091), there might also be other important differences between the regions than shown in the identified predictors.

As in all studies based on interviews, the physicians’ answers might also have been influenced by memory gaps, errors or social desirability. This relates, in particular, to the assessment of the consultation reasons and the service spectrum which were mainly retrieved from memory and which could not be verified through a clinical audit of records. It should also be noted that 12 of the 211 GP interviews (5.7%) were completed by telephone which might have impaired detail of the GPs’ answers. However, using interview data facilitated a series of analyses that would not have been possible on the basis of routine data. For example, services paid by flat rate could be depicted individually in our study and reasons for consultation, such as “social problems” which are difficult to code in ICD-10, could be recorded. Another limitation is the fact that the recruitment area has been restricted to northern Germany and the results therefore do not necessarily represent the remainder of Germany. As this was an observational study with multiple outcomes, we were unable to carry out a sample size calculation and therefore might have missed some differences between the regions due to limited statistical power.

### Comparison with literature and discussion of results

Our study suggests that the frequency of the individual reasons for consultation differ between urban and rural areas. Compared to urban areas, fewer consultations for “social problems” were reported in rural areas indicating that GPs in urban areas probably had to provide more psychosocial support for their patients than GPs in rural areas. This could be the result of regionally different housing situations. One of the results of the EU contribution to the World Mental Health Surveys Initiative was that life in urban areas seemed to increase the risk of affective disorders [17]. An analysis of statutory health insurance physicians’ billing data also reported on the increased prevalence of depressive disorders in German core cities [4].

“Acute infections of the respiratory tract” had also been reported less frequently in rural areas than in environs. This may be an indication that social medical tasks, such as issuing a statement of illness, are given higher priority in districts with a higher degree of urbanisation than rural areas. This point had already been stated by some participants of our preliminary qualitative study by saying that GPs in urban areas were often only perceived as medical service providers or referrers to other specialists [9].

In Germany, patients are permitted to consult specialists without the referral from a GP. In our study, GPs in rural areas more often reported reasons for consultation for which urban patients probably would have directly used the services of a specialist, primarily concerning orthopaedics, dermatology and otorhinolaryngology. This finding might be explained by the different regional availability of physicians in outpatient care. On average, rural areas included in this study only showed a lower density of ca. 10% of physicians in primary care but a 40 to 50% lower density of ophthalmologists, ENT specialists and orthopaedists in comparison to urban areas or environs [16]. The low availability of specialists in rural areas has been widely discussed in literature [16, 18].

GPs in rural areas reported to treat a larger number of patients and to offer a broader spectrum of services than GPs in urban areas. Other studies showed the same results. Steinhäuser et al., for example, reported that GPs in rural areas carried out a broader range of procedures, particularly those usually playing a role in specialist care [10]. As early as 1998, Boerma et al. investigated regional differences in the spectrum of services provided by GPs from 30 European countries and described a more comprehensive range of services by primary care practices in rural areas and indicated a smaller role as gatekeeper of the inner city primary care practices [19]. Mehring et al. also described a higher ratio of coordinated care by GPs of patients from rural areas [20]. Additionally, a study on the treatment of bowel cancer highlighted a greater involvement of GPs from rural areas in coordinating care as well as clinical and psychosocial support for patients in rural areas [21].

Our study also showed differences in the type of individual services. GPs in rural areas, for example, spent less time on preventive medicine than GPs in urban areas. This might indicate a shortage of preventive services in rural areas. The more frequent mentioning of “local injection / infiltration” or “urine analysis” in rural than in urban areas or environs might be interpreted as an indication that classic somatic primary care services had been carried out more frequently in rural than in urban areas or environs. House calls had also been reported more frequently in rural than in urban areas. Pochert et al. also described this regional difference in their study on the house call workload of GPs in Germany. Their study showed an average number of 16.5 house calls per week in rural areas whereas only 12.5 house calls per week were carried out in urban areas [22].

Regarding the level of services, GPs in urban areas placed greater emphasis on psychosocial support. A study by Görig et al. showed that GPs in rural areas offered fewer lifestyle consultations for preventing cardiovascular disorders than GPs in cities [23]. As already mentioned, there is a higher prevalence of psychological disorders, such as depression or anxiety disorders in urban than in rural areas [24, 25]. This may lead to the conclusion that GPs from urban regions have to provide psychosocial services for the care of these patients more frequently. Likewise, access to psychotherapy and psychosocial support in urban areas is also generally better than in rural areas. Just under 25% of the German population lives in cities compared to almost 50% of Germany’s psychotherapists who practise in cities [26]. However, GPs from urban areas still spent more time on psychosocial support than GPs from rural areas. This might indicate a underassessment of psychosocial problems in rural areas.

## Conclusion

Our study pointed out a number of regional differences that might be relevant for health care planning, particularly regarding the services provided in rural and urban areas. It suggests that GPs in rural areas more frequently provide services that are usually part of specialist care. There are many approaches that may help motivating young physicians to work in rural areas, such as improving rural infrastructure. But as there are generally too few young physicians, other strategies should also be taken into consideration, eg, telemedicine, mobile practices or delegation of medical services. In our study, GPs in rural areas mostly worked in individual practices while GPs in regions with a higher degree of urbanisation more often worked in group and joint practices or medical care centres. This might be the result of the lower mean age of GPs working in urban areas as practices with a larger staff of physicians usually provide better and more family-friendly working conditions, which might be more appealing to young physicians.

Another finding that might be relevant in this context was the fact that rural GPs’ broader service spectrum was statistically explained by a higher level of medical training. On the one hand, this result might imply that GPs from rural areas compensated for specific needs of the regional patient population by completing relevant advanced medical training. On the other hand, this might also imply that predominately GPs with advanced medical training were drawn to rural areas, eg, because they felt better prepared for the local conditions. Further research is needed to clarify the role of medical training for interventions addressing the shortage of rural GPs.

## Supplementary information

**Additional file 1: Table S1.** planned and completed sample size in the region “urban areas “. **Table S2.** planned and completed sample size in the region “environs”. **Table S3.** planned and completed sample size in the region “rural areas”. **Table S4.** Association between region and number of different reasons for consultation: results of a multivariate linear regression adjusted for random effects on the levels of German federal states and administrative districts within the federal states (*n* = 203).

## Data Availability

The datasets analysed during the current study are not publicly available as data sharing with other researchers was not part of patients’ informed consent.

## Abbreviations

EDP: Electronic data processing
ENT: Ear, nose, and throat
GP: General practitioner
ICPC-2: International Classification of Primary Care
