# Supplementary material for: Regional differences in reasons for consultation and general practitioners’ spectrum of services in northern Germany – results of a cross-sectional observational study
Source: BMC Fam Pract. 2020 Jan 31;21:22. doi: 10.1186/s12875-020-1093-6 (PMC6995090; doi:10.1186/s12875-020-1093-6)
Supplement: Supplementary file 1 — Additional file 1: Table S1. planned and completed sample size in the region “urban areas “. Table S2. planned and completed sample size in the region “environs”. Table S3. planned and completed sample size in the region “rural areas”. Table S4. Association between region and number of different reasons for consultation: results of a multivariate linear regression adjusted for random effects on the levels of German federal states and administrative districts within the federal states (n = 203). [file 12875_2020_1093_MOESM1_ESM.pdf]

Additional file 1

Table S1. planned and completed sample size in the region “urban areas “

Administration district	Inhabitants	GPs planned	Recruited GPs	Participating GPs
**Bremen**				
- Bremen Centre	18,231	1	2	**2**
- Bremen East	223,753	6	2	**1**
- Bremen North	97,460	3	5	**5**
- Bremen South	126,710	3	2	**2**
- Bremen West	91,310	2	5	**4**
- Bremerhaven	114,025	3	3	**2**
**Hamburg**				
- Hamburg Altona	267,058	7	11	**10**
- Hamburg Bergedorf	124,998	4	4	**3**
- Hamburg Centre	296,410	8	10	**8**
- Hamburg Eimsbüttel	258,865	7	2	**2**
- Hamburg Harburg	160,211	4	5	**4**
- Hamburg North	302,242	8	10	**8**
- Hamburg Wandsbek	424,146	11	13	**10**
**Kiel**	246,306	7	4	**3**
**Lübeck**	216,253	6	4	**4**
		80	82	**68**

Table S2. planned and completed sample size in the region “environs”

Administration district	Inhabitants	GPs planned	Recruited GPs	Participating GPs
**Altmarkkreis Salzwedel**	86,164	2	4	**3**
**Delmenhorst**	76,323	2	1	**0**
**Diepholz**	16,692	1	0	**0**
**Harburg**	248,122	7	9	**7**
**Herzogtum Lauenburg**	192,999	5	7	**3**
**Neumünster**	79,197	2	3	**3**
**Nordwestmecklenburg**	156,270	4	6	**5**
**Oldenburg**	128,608	4	2	**2**
**Osterholz**	113,579	3	0	**0**
**Ostholstein**	199,574	5	5	**4**
**Pinneberg**	307,471	8	12	**10**
**Plön**	128,304	4	4	**3**
**Rendsburg-Eckernförde**	270,378	7	11	**10**
**Segeberg**	267,503	7	8	**7**
**Schwerin**	96,800	3	3	**3**
**Stade**	200,054	5	13	**7**
**Stormarn**	239,614	7	8	**6**
**Verden**	134,645	4	5	**3**
		80	101	**76**

Table S3. planned and completed sample size in the region "rural areas"

County*	Inhabitants	GPs planned	recruited GPs	Participating GPs
**Celle**				
- City of Celle	69,748	2	1	**0**
- rural area	108,223	4	8	**4**
**Cuxhaven**				
- City of Cuxhaven	48,264	2	1	**0**
- City of Geestland	30,936	1	0	**0**
- rural area	118,903	4	5	**5**
**Dithmarschen**				
- City of Heide	21,422	1	0	**0**
- rural area	111,495	4	10	**7**
**Gifhorn**				
- City of Gifhorn	41,905	1	1	**1**
- rural area	132,300	5	3	**1**
**Heidekreis**				
- City of Soltau	21,414	1	1	**0**
- City of Walsrode	23,219	1	3	**2**
- rural area	95,631	3	5	**4**
**Ludwigslust-Parchim**	214,113	8	8	**5**
**Lüchow-Dannenberg**	50,128	2	9	**8**
**Lüneburg**				
- City of Lüneburg	74,072	3	4	**2**
- rural area	106,647	4	5	**4**
**Nienburg**				
- City of Nienburg	31,193	1	2	**2**
- rural areas	89,439	3	2	**2**
**Nordfriesland**				
- City of Husum	22,430	1	1	**1**
- rural area	141,260	5	7	**7**
**Rotenburg (Wümme)**				
- City of Rotenburg	21,392	1	0	**0**
- rural area	141,861	5	4	**3**
**Schleswig-Flensburg**				
- City of Schleswig	24,266	1	0	**0**
- rural area	172,573	6	4	**3**
**Steinburg**				
- City of Itzehoe	31,771	1	2	**2**
- rural area	99,686	4	4	**3**
**Uelzen**				
- City of Uelzen	33,782	1	0	**0**
- rural area	59,349	2	5	**4**
**Wesermarsch**				
- City of Nordenham	26,325	1	0	**0**
- rural area	62,914	2	2	**2**
		80	97	**72**

* stratified by cities of more than 20,000 inhabitants and rural areas

Table S4. **Association between region and number of different reasons for consultation: results of a multivariate linear regression adjusted for random effects on the levels of German federal states and administrative districts within the federal states (n=203)**

	**Model 1**		**Model 2**		**Model 3**	
	**ß (95% CI)**	**p**	**ß (95% CI)**	**p**	**ß (95% CI)**	**p**
Region						
- urban areas vs. rural areas	-0.51 (-5.81 to 4.79)	0.850	0.62 (-4.70 to 5.94)	0.820	2.33 (-3.23 to 7.88)	0.412
- environs vs. rural area	0.56 (-4.61 to 5.73)	0.831	1.86 (-3.36 to 7.07)	0.485	2.74 (-2.50 to 7.97)	0.306
Age of the physician (in years)	0.03 (-0.23 to 0.29)	0.803	0.16 (-0.11 to 0.43)	0.246	0.16 (-0.11 to 0.43)	0.253
Gender of the physician: male vs. female	0.15 (-4.36 to 4.65)	0.948	-0.34 (-4.76 to 4.08)	0.880	-1.38 (-5.87 to 3.12)	0.548
Postgraduate medical specialist training						
- none (general practitioner)			-11.26 (-23.75 to 1.23)	0.077	-11.55 (-24.10 to 1.01)	0.071
- general medicine			1.57 (-7.12 to 10.27)	0.723	1.22 (-7.49 to 9.92)	0.784
- internal medicine			-1.92 (-9.83 to 5.99)	0.634	-1.79 (-9.69 to 6.10)	0.656
Number of areas of advanced medical training			0.06 (-1.29 to 1.42)	0.927	0.04 (-1.32 to 1.39)	0.958
Type of practice						
- group practice vs. private practice					-4.10 (-13.23 to 5.03)	0.378
- joint practice vs. private practice					-0.01 (-4.56 to 4.53)	0.996
- medical care centre vs. private practice					-4.40 (-21.62 to 12.82)	0.617
Number of treated patients (per 100 patients every month)					0.02 (-0.00 to 0.04)	0.088
